# Multigenerational social mobility and depressive symptoms among middle-aged and older Chinese

**DOI:** 10.1093/geroni/igaf122.3014

**Published:** 2025-12-31

**Authors:** Yi Huang, Peiyi Lu, Chihua Li

**Affiliations:** The University of Hong Kong, Hong Kong, China (People’s Republic); The University of Hong Kong, Hong Kong, China (People’s Republic); University of Macau, Macao, Macao, China (People’s Republic)

## Abstract

Intergenerational social mobility is associated with mental health in older age. However, most research focused on mobility across two generations and used a single indicator (e.g., education). This study explored the pattern of social mobility across three generations using four socioeconomic indicators and examined their association with depressive symptoms among middle-aged and older Chinese. Respondents (aged ≥ 45) from the China Health and Retirement Longitudinal Study were included (N = 3,314). Information regarding the hukou, education, occupation, and Communist Party membership of the respondents’ parents, the respondents themselves, and the respondents’ children was retrieved. Latent class analysis identified groups with similar social mobility patterns across three generations. Mixed-effects models examined the association of the class memberships with depressive symptoms trajectory in 2011-2018. Five classes were identified: Class 1 (37.90%) showed a slow rise in social status while remaining rural hukou; Class 2 (26.69%) exhibited a steady rise in social status while remaining rural hukou; Class 3 (24.11%) experienced a rapid rise in social status and hukou converting from rural to urban; Class 4 (1.12%) showed a steady rise in social status while remaining urban hukou; Class 5 (7.18%) maintained a high social status and remained urban hukou. Compared to the “Slowly rising rural”, steadily or rapidly rising or stably high social status was associated with a lower risk of depressive symptoms at baseline. This study underscores the disadvantaged status of individuals with rural hukou and slow multigenerational upward social mobility, calling for more targeted mental health support for them.

